# Charles Hamilton Fasson

**Published:** 1892-12-17

**Authors:** 


### HOSPITAL WORTHIES.

### CHARLES HAMILTON FASSON, M.D.

Another hospital worthy, whose great services to the cause of efficient hospital administration were far too little known outside his immediate sphere of work, has, we regret to say, passed away. Deputy Surgeon-General Fasson for twenty-seven years was attached to the Army Medical Service, during which period he passed through the Indian Mutiny, and was subsequently Registrar in the Herbert Hospital at Woolwich. When it was determined to rebuild the Royal Infirmary, Edinburgh, in 1870, it became necessary to look out for a medical superintendent of experience and ability to take charge of the administration. Mr. Fasson was selected after a keen competition from a large number of candidates, and became Superintendent of the Edinburgh Royal Infirmary in 1871. We had occasion to inspect the old Infirmary before it had been determined to rebuild it upon a new site. Few who have not seen the actual state of affairs to be met with at this institution in those days can credit the abuses which prevailed in nearly every department. The buildings were terribly insanitary; the management, if such it can be called, was sadly behind the times, and the condition of the nursing and the internal discipline was so bad as to make one wonder how it could have been allowed to continue for the lengthened period during which it prevailed. No good purpose would be served by our recalling the terrible facts at the present day, but it is desirable to mention the circumstances of those times in order to do justice to the services rendered by the late Mr. Fasson. Mr. Fasson had the singular good fortune to be associated with Miss Pringle, the Lady Superintendent of nursing, and her assistant, Miss Spencer, who succeeded Miss Pringle on the latter's appointment to St. Thomas's Hospital. We may say in passing that the nursing world has probably never produced two abler women than Miss Pringle and Miss Spencer, and that few, if any, of the many able ladies who now devote their life to nursing can equal, much less surpass, the services rendered at Edinburgh by these two ladies. Miss Spencer's great ability and entire devotion must impress any visitor to the Royal Infirmary at the present time, and as they go through the wards and inspect the new Nurses' Home they will meet with practical evidence, in every direction, of the efficiency of the system which regulates nursing matters at this institution, and of the ability and skill displayed by those in authority.

Mr. Fasson set to work at once to introduce needful reforms. He was endowed with singular ability and tact, and his grace of manner, gentleness, and amiability proved irresistible in practice. The most prejudiced and determined opponent of reform, when brought face to face with Mr. Fasson, found it impossible to persist in opposing the changes, which he was able to show would produce results well calculated to enhance the fame and reputation of the Royal Infirmary. Mr. Fasson is the author of a system of internal administration, directed to the enforcement of economy in all departments, which we venture to say is unequalled for the efficiency of its results, and for the general acceptance which it has received at the hands of the medical staff, the resident officers, and the Board. He it was who hit upon the plan of preparing forms, which brought out clearly the expenditure upon the patients of every member of the medical staff, and which showed in detail how much was spent on each item which goes to make up the dietary of a hospital patient. Periodically, these startling figures were brought together in one simple form, a copy of which was sent out to every member of the staff and submitted to the Board. When a physician finds, from the figures that confront him, that his patients are consuming four times the amount of alcohol, or twice as many eggs or expensive diets of a special kind, as those of his colleagues, it necessarily follows that a close inquiry is made for the sake of his own reputation, and that, in the result, a considerable reduction in the expenditure follows without injury to the patient or interference with his treatment. Similarly, the surgeons were confronted with the actual cost of the dressings used in the treatment of their cases, and they, in turn, made close inquiries, and enforced a careful use of expensive dressings, which stopped waste and prodigal use, and so brought down the expenditure in a department, which had heretofore spent without any regard to such practical considerations. Mr. Fasson further made it his business to see that every member of the resident staff, from the humblest to the highest, was provided with suitable accommodation, and an abundance of good food, well cooked and tastily served. To the nurses Mr. Fasson was a father, who combined loving care with shrewd discernment and the enforcement of necessary discipline and order, which, thanks also to Miss Pringle and Miss Spencer, have made the nurses who have been trained at the Royal Infirmary, Edinburgh, amongst the most practical and useful to be met with in this country to-day. Mr. Fasson was devoted to his work. He found that the amount of it was so large, and the duties connected with the care of the Royal Infirmary so absorbing, that he unfortunately never wrote anything upon a subject which he had thoroughly mastered and in which he might claim to be a leading authority. It is a great fact, as the Lords' Committee recently pointed out, that the voluntary hospitals of this country are able to command for a surprisingly moderate salary the services of able and devoted men, who make it their business to be thoroughly acquainted with everything which affects the best interests of the institution, the management of which has been placed in their hands. Among such men the late Mr. Fasson held a foremost place, and his death has caused a vacancy which it will be difficult to fill, whilst the remembrance of his services at Edinburgh will long live in the memory of all who love the Royal Infirmary, and desire to see it occupy a foremost place amongst the hospitals of the British Empire.

During the last ten years at least, Mr. Fasson's health was far from strong, and it pleased Providence to visit him with a succession of domestic troubles so distressful as to make one wonder how the man could continue at his post. No doubt his heart was in his work, and as these troubles came upon him one by one, they caused him to cling with increasing devotion to the interests of an institution which he loved so well. His courtesy, his amiability, his powers of conciliation, and force of character, constitute a monument, which is founded on the solid facts that he has brought order out of chaos, and made the Royal Infirmary what it is to-day. It was the special wish of Miss Spencer and the nursing staff, and his own earnest purpose also, to be present at the opening of the magnificent Nurses' Home, which has just been completed. Providence ordered otherwise, however, but those whom he has left behind, will, no doubt, with full hearts and grateful memories, when the ceremony takes place, recall all that Mr. Fasson has done, not only for the Royal Infirmary, but for each individual member of the staff, of which he was for twenty-one years the beloved chief.

At a recent meeting of the Board of Directors, the minutes which follow, commemorating the services of the late Deputy Surgeon-General Fasson, were unanimously approved. Such deserved appreciation of the services of a good man to a great institution has seldom been more gracefully recorded than those of Mr. Fasson are in the following resolutions :—

The managers record their sense of the great loss sustained by the Royal Infirmary in the death of Mr. Fasson. Appointed Superintendent of the Infirmary in 1871, he brought to the work of his office a combination of gifts and experience which singularly fitted him for its important duties, and these he has throughout discharged with eminent ability, unwearied assiduity, and well-deserved success. That work included the difficult and responsible task of transferring the Infirmary to the present buildings, and the reconstruction to a large extent of its administrative department. Yet by no startling change, but by the steady introduction of improved methods of which he had satisfied himself, was reform carried out. Ever intent on schemes for securing smoother and more efficient working of the hospital, he gladly welcomed and adopted suggestions from any quarter which tended in this direction. Firm in his administration, exact in his demands upon himself, and expecting equal fidelity and efficiency in others, his relations to all whose duties he had to superintend were distinguished by great consideration and courtesy. It was in no small measure due to the practical sagacity and foresight of Mr. Fasson that the fever wards of the Infirmary were in 1885 transferred to the city authorities—a change which, as the event has proved, has been greatly to the public advantage. The nursing department of the Infirmary underwent entire reconstruction during Mr. Fasson's tenure of office. This was a work in which he took a profound interest, and the high position which the Royal Infirmary now holds among hospitals as a training school for nurses owes much to his ever-ready and hearty sympathy and co-operation. In providing the nurses with adequate accommodation Mr. Fasson warmly interested himself, and the building of the new home, now nearly completed, was undertaken by the managers largely upon his strong recommendation. The arrangements for its furnishing and occupation were engaging his attention in the last days of his life. The tone which happily pervades every department of the noble institution with which Mr. Fasson was so long and honourably connected is undoubtedly to be ascribed to the influence which he quietly but most effectively exercised. Of Mr. Fasson's personal qualities it is impossible to speak too highly. His gentleness, his perfect fairness, his unvarying courtesy, and his tenderness of heart endeared him to all by whom he was known. These qualities ever worked outward from what were the dominating influences of his life—high principle and Christian character. The managers share deeply in the feeling of personal loss which Mr. Fasson's death has evoked in all who in any capacity minister within the walls of the infirmary, and they are keenly sensible of the very difficult duty which, through his death, has devolved upon them of finding a superintendent worthy to succeed him. They resolve that an extract of this minute shall be sent to his afflicted family, with the assurance of the managers' heartfelt sympathy with them in their bereavement.

